# Biomedical Application of Polymeric Materials

**DOI:** 10.3390/polym17172401

**Published:** 2025-09-03

**Authors:** Andreea-Teodora Iacob

**Affiliations:** Department of Pharmaceutical and Therapeutic Chemistry, Faculty of Pharmacy, University of Medicine and Pharmacy “Grigore T. Popa” of Iasi, 16 University Street, 700115 Iasi, Romania; andreea.panzariu@umfiasi.ro

## 1. Introduction

The term ‘polymer’ was first introduced by Swedish chemist Jöns Jacob Berzelius to describe macromolecules consisting of significant repeating structural units [1]. In polymer and materials research, a distinction is maintained between bio-macromolecules and biopolymers, such as polysaccharides or proteins, and wholly synthetic polymers, such as polyethylene or polyethylene terephthalate. The disparity mostly arises from their origins and production methods; biopolymers are derived from natural sources like plants or animals, whereas synthetic polymers are produced from petroleum-based monomers. Both bio- and synthetic polymers have significantly influenced our lives over the past century and are crucial in sectors such as textiles, healthcare, food packaging, and construction [2]. Advanced polymer materials are essential elements in the global economy and high-end industry [3]. Numerous industries, including packaging, cosmetics, sutures, dentistry (including prosthetic teeth and filling materials), material manufacture, and the food and beverage sectors, have significantly embraced polymer-based materials [4].

Currently, it is well acknowledged that polymers serve as the foundation for a diverse array of materials. These materials are extensively employed in biomedicine owing to their multifaceted characteristics. Biodegradable polymers, capable of natural decomposition via biological processes, are being evaluated for various applications. Biodegradable polymers are acknowledged as superior materials for biomedical applications due to their remarkable biocompatibility. Biomaterials are a multidisciplinary research domain that integrates material science with medical applications, emphasizing mechanical qualities and biocompatibility [1,5].

The versatility of chemistry facilitates the development of polymers with diverse mechanical and physical properties, rendering them particularly advantageous in biomedical research. The intrinsic capacity of biomaterials to break down and be assimilated or eliminated by the body without surgical intervention renders biodegradable polymers especially advantageous. Due to their low immunogenicity and biocompatibility, natural source polymers are commonly employed in biomedical applications [6]. They are selected for medicinal applications due to their minimal likelihood of inducing allergic responses. Synthetic polymers offer alternatives for persons susceptible to allergies, as they reduce immune reactions and chronic inflammation [4]. Biomedical polymer materials can efficiently encapsulate a large variety of small molecules, proteins, and functional nucleic acids by surface modification. Biopolymeric materials serve a vital function as scaffold materials in tissue engineering and regenerative medicine, facilitating cell adhesion, proliferation, and differentiation to repair or replace damaged tissues [7,8].

The methods employed in the preparation of polymer matrices significantly affect the properties of the polymer. Preparation processes affect factors like the stability, thermal conductivity, mechanical properties, barrier properties, and surface morphology of polymer matrices [9]. Selecting preparation processes according to the intended application guarantees the optimal performance of polymer matrices, facilitating the development of innovative materials with customized features [8]. Hyper-branched polymers (HPBs), a category of extensively branched three-dimensional (3D) macromolecules, exhibit attributes like ease of chemical manipulation and multi-cavity architectures. By means of suitable design, they can meet the specifications of biomedical materials in medical applications [10].

The purpose of this Special Issue is to describe the latest discoveries and research regarding polymeric materials that have applicability in the biomedical and pharmaceutical fields. This Special Issue includes 21 papers (of which 15 are articles and 6 are reviews) that will be briefly described below in order to spark readers’ interest in studying them in detail.

## 2. An Overview of the Published Articles

**Figure 1:**
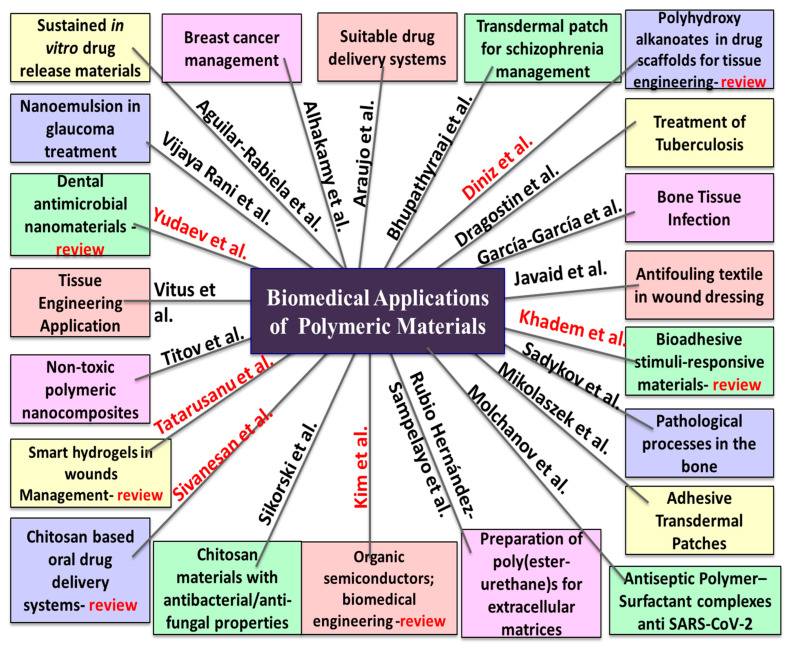
Schematic representation of the subject of each article published in the Special Issue “Biomedical Application of Polymeric Materials” found in the list of contributors (1–21), where the authors in red contributed with a review type of article.

The articles published in this Special Issue, which are on such a wide-ranging topic, cover a broad spectrum of biomedical applications, which are summarized in Figure 1.

In the context of their research, Aguilar-Rabiela et al. articulate a comparison between the astaxanthin (ASX) release profile from MBGNs (mesoporous bioactive glass nanoparticles) and from the composite microspheres PHBV/MBGNs (where PHBV stands for poly (3-hydroxybutyrate-Co-3-hydroxyvalerate)). Because of the regulated release of both ASX and advantageous ions from MBGNs as well as dissolving byproducts from the MBGN/PHBV composite material, it was reported that this composite approach increases cell activity. It was shown that the composite material demonstrated an extended release of ASX for up to 80 h, in contrast to the 40 h observed with the MBGN/ASX. The fluorescent cell morphology measurements and WST-8 cell viability results indicated that the variation in release behavior influenced NIH-3T3 cell viability over the initial 72 h of incubation. The results indicated that the release behavior of the composite microspheres can be adjusted by altering the MBGN content within them. Both the MBGN/ASX and composite microspheres showed optimal potential for controlled phytotherapeutic delivery required for particular applications.

The investigation carried out by Alhakamy et al. reveals the outstanding development of scorpion venom (SV)-functionalized quercetin (QRT) phytosomes for breast cancer treatment, accompanied by an anti-proliferative and anti-apoptotic assessment using the human breast cancer MCF-7 cell line. The improved QRT-SV phytosome formula demonstrated a markedly enhanced expression of p53 mRNA, Bax, Bcl-2, and caspase-9 and significantly diminished NF-κB and TNF activity in comparison to both the basic formula and QRT alone.

Through their research, Araújo et al. shed light on the optimization of chitin–glucan complex (CGC) hydrogels and their evaluation in terms of drug loading (caffeine) and release ability. The loaded hydrogels exhibited enhanced rheological and mechanical characteristics, with caffeine release profiles adhering to a Fickian diffusion mechanism in PBS solution and a non-Fickian diffusion in 0.9% NaCl solution. This study shows that CGC may be transformed into hydrogels by a straightforward technique, and the resultant structures exhibit appropriate characteristics for application as drug delivery vehicles.

The research presented by Bhupathyraaj et al. investigates the impact of polymers and permeation enhancers (1,8-cineole, linalool, and DMSO) on the release of quetiapine fumarate (QTP-F) from transdermal patches via a dialysis membrane. The best QTP-F formulation proved to be the F2 transdermal patch, utilizing a 50:250 ratio of the polymers PVP K30 (polyvinylpyrrolidone K30) and HPMC K100 (hydroxypropyl methylcellulose K100) in conjunction with the natural permeation enhancer 1,8 cineol. The stability research results indicated no substantial alteration from its initial state over a three-month period at both temperatures (room temperature and 40 °C). The F2 was reported to successfully attain cost-effectiveness, extended-release capabilities, reduced dosage, and diminished administration frequency, potentially enhancing patient adherence to the schizophrenia treatment.

Diniz et al. offer an in-depth analysis of the contemporary biotechnological utilizations of PHA (poly-hydroxy-alkanoates) in the biomedical field in a 17-page review. The review emphasizes the recent research concerning various PHA in the biomedical field, with the objective of elucidating the approaches employed and highlighting the biopolymer’s versatility. Their use in drug carriers is prevalent, owing to their biocompatibility, their degradation yields products that are natural to the body, and their biodegradability facilitates the direct application of drugs in targeted tissues. This enhances the compound’s applicability while mitigating the inherent toxicity of certain drugs, rendering them more appealing than alternative polymers due to their ability to be recognized by the human body as degradation products that are naturally eliminated. Consequently, these polyesters are demonstrated as a feasible alternative to petroleum-derived plastics, mitigating the environmental effect associated with them. The economic impediment posed by the elevated production costs of these polymers remains a significant challenge, as evidenced by this review. Therefore, the investigation showed an approach that integrates the bioremediation of wastewater and optimizes the utilization of microorganisms that necessitate lower cultivation expenses, such as microalgae, which can thrive in wastewater and sludge while requiring fewer nutrients due to their autotrophic nature.

In the study by Dragostin et al., the authors explore the in vitro and in vivo evaluation of chitosan (CS) microparticles loaded with isoniazid (INH) derivatives for tuberculosis (TB) treatment. Their work demonstrated the benefits of microencapsulating INH derivatives on liver damage caused by anti-TB medication through mitigating the hepatotoxicity of the compounds and facilitating the correction of cell necrosis and microvesicular steatosis in contrast to their administration in suspension. The findings indicated a substantial decrease in tissue modifications, the elimination of cellular necrosis and microvesicular steatosis, and reduced levels of alkaline phosphatase and the liver enzymes TGO and TGP when employing encapsulated medication formulations.

The article written by García-García et al. provides insight into the in vivo response of a polymeric construct composed of PLGA, poly(lactic-co-glycolic acid), within the solid phase of Palacos R^®^ combined with antibiotics for the treatment of methicillin-resistant staphylococcal infections such as daptomycin, vancomycin, and/or linezolid. The conducted experiments confirmed that the addition of PLGA microspheres in Palacos R^®^ bone cement and the incorporation of the antibiotic daptomycin along with vancomycin enhance the tissue response to bone infection. Nevertheless, the antibiotic linezolid combined with vancomycin yielded suboptimal outcomes in our model, irrespective of the cement type employed.

Javaid et al. provide a detailed report on a novel method for producing nanoencapsulated cotton textile fabrics by integrating antifouling functional group finishing, utilizing a layer-by-layer technique through the alternating antifouling polymeric formulations (APF) and dip coating of oppositely charged polyelectrolyte solutions. The nanoencapsulated finished antifouling cotton textile fabric may be utilized in diverse industrial applications, especially in wound dressings, for the prevention of skin infections.

In their 37-page review, Khadem et al. focus on an extensive examination of advanced smart adhesives, their constraints, and the prospective trajectories and obstacles for the forthcoming generation of smart bioadhesives. Initially, the primary requirements for bioadhesives and classifications of smart bioadhesives were examined. Secondly, the use of smart bioadhesives in diverse applications, including tissue engineering, wound healing, and medication administration, was investigated. The final section addressed the shortcomings and difficulties of the present research, as well as the potential paths for smart bioadhesives.

The review conducted by Kim et al. provides an overview of organic semiconductors in biomedical applications, encompassing their characteristics, the device architectures, fabrication techniques, and uses in documented organic material-based healthcare devices. The assessment of material properties and fabrication advantages indicates that organic semiconductors possess significant promise for biomedical applications, particularly in flexible and wearable medical devices. Due to the superior mechanical characteristics of organic semiconductors, inherently flexible electronic devices can be created. This review also highlights the limitations of these organic semiconductors, such as unsatisfactory robustness which hinders their commercialization, reduced reliability attributable to the erroneous transmission of information and sensory signals over an extended duration, and the focus on biodegradability and biocompatibility, important challenges that must be addressed.

In the findings published by Mikolaszek et al. in this Special Issue, attention is drawn to a comparative investigation of pressure-sensitive adhesive polymers (PSA), specifically silicone and acrylate matrices (DuroTak^®^ 387-2287, DuroTak^®^ 87-4098, DuroTak^®^ 87-2852, Bio-PSA MD7-4502, and SoftSkinAdhesive MG7-9850) designed for transdermal patch formulations. Three active pharmaceutical ingredients (APIs) (indomethacin, cytosine, and testosterone) with varying lipophilicity and transdermal transport capabilities were examined. While optical microscopy can assess the API state in the matrix, as well as the morphology, distribution, and particle size of the undissolved fraction, surface-focused SEM offers supplementary insights into the 3D positioning of the API, which may aid in elucidating the release kinetics.

Molchanov et al. describe the antiseptic polymer–surfactant complexes incorporating the cationic disinfectant cetylpyridinium chloride (CPC) exhibiting prolonged efficacy against SARS-CoV-2. The release rate of the CPC from the oppositely charged polymer gels (copolymers of (i) vinyl pyrrolidone and sodium methacrylate; (ii) acrylamide and sodium 2-acrylamido-2-methylpropane sulfonate; and (iii) acrylamide and sodium methacrylate) can be adjusted over a considerable range by altering the disinfectant concentration, swelling degree, cross-linking extent of the gel, and the content/type of anionic repeat units in the polymer matrix. Polymer–surfactant complexes were shown to decrease SARS-CoV-2 titer by seven orders of magnitude within just 5 s. The complexes maintained significant virucidal efficacy against SARS-CoV-2 for a minimum duration of one week.

In the study reported by Rubio Hernández-Sampelayo et al., emphasis is placed on the synthesis of a novel aromatic isocyanate derived from diamino-PABA (diamine 1,3-propanediol bis(4-aminobenzoate)) and the characterization of linear polyurethanes (PUs) obtained from it. Unhydrolyzable 1,4-butanediol (BD), together with hydrolyzable N,N-ethylene-bis(6-hydroxycaproamide) (EDA2 CL), were used as chain extenders, whereas polycaprolactones (PCLs) of different molecular weights were used as soft segments. Cell survival after 28 days for all poly(ester-urethane)s containing isoPABA motifs and EDA-2CL chain extenders exceeded 80%, indicating that these polymers were non-toxic. Furthermore, Alamar Blue data demonstrated favorable cell adhesion and cytotoxicity on the surfaces of these non-toxic biodegradable polyurethanes.

In their study, Sadykov et al. address the development of cryogel composite materials derived from polyvinyl alcohol and calcium phosphates, together with the examination of their physicochemical, functional, and mechanical attributes. The investigation into monocyte viability revealed that approximately 60–80% of the cells persist in the presence of pure HA, while the survival rates in the presence of pure PVA and composites are equivalent to the control sample. The proposed materials can be deemed biocompatible, as immune response monocytes exhibit high viability in their presence. This material can be utilized in biomedical applications to fill holes created by pathological processes in bone, as well as between the bone and the mechanically loaded implant.

Sikorski et al. examine the way in which the acid salts of chitosan (CS) can influence the antibacterial/antifungal potential of CS-based materials. For this study, different acid-based CS nonwovens (acetic, butyric, formic, hydrochloric, propionic, valeric) were taken into account in the analysis. ^1^H-NMR spectroscopy and FTIR-ATR spectroscopy were employed to verify the integration of acid groups with the amino groups of chitosan. The developed materials underwent microbiological testing. Each modified material was inoculated with bacteria. Antimicrobial activity was detected in CS salts with acetic acid and hydrochloric acid. A decrease in the quantity of bacterial cells was noted for the *S. aureus* strain treated with CS salt modified with 10% acetic acid in ethanol. The antibacterial efficacy of CS salts increased with the proportion of acid salts present on the solid material’s surface, resulting in a reduction or absence of bacterial colonies. No decrease in growth was noted for the *E. coli* strain. The CS samples were either inactive or entirely eradicated the bacterial cells. The toxicity to human erythrocytes was also examined. The toxicity data for human red blood cells, quantified as a percentage of hemolysis, indicate significantly greater toxicity in the samples that were not rinsed. The maximum toxicity recorded was 21.18% for the sample subjected to acetic acid treatment. A total lack of hemolysis was noted in the samples treated with valeric acid and formic acid.

The paper written by Sivanesan et al. discusses the current state of knowledge on examining the diverse new CS compounds available for drug delivery applications. A complete evaluation has been conducted on the milestones attained by applying CS derivatives in oral medication administration. The absence of utilizing various CS derivatives for oral medication administration has been emphasized. Additionally, potential explanations for the disparity in the utilization of distinct CS derivatives for oral medication administration were examined. The potential achievements attainable by using available resources were discussed in the future perspectives section.

The review realized by Tatarusanu et al. focuses on the recent literature concerning polymer-based wound dressings as modern approaches in wound healing. This overview succinctly outlines the physiopathology and healing mechanics of chronic wounds, along with contemporary therapy strategies. In this study, the rationale for employing standard and smart hydrogels (SHs) in wound healing was also discussed, along with current research trajectories aimed at generating SHs with new characteristics while also addressing their limitations and prospects for industrial-scale production.

In the findings published by Titov et al., attention is drawn to the morpho-functional condition of the sensori-motor zone of the cerebral cortex and the hepato-renal system following the acute and sub-acute administration of silver selenide nanoparticles (AgSe NPs) encapsulated in a natural arabino galactan (AG) polymer. The investigation of acute toxicity, conducted through the oral administration of nanocomposites at a dosage of 2000 mg/kg, has demonstrated that the material in question is classified as a low-toxicity substance within the fifth hazard class. Sub-acute oral administration of nanocomposites at a dosage of 500 g/kg results in minor alterations in the brain tissue and liver of experimental subjects, suggesting the onset of compensatory adaptive responses. The Shumlyansky–Bowman chamber in the kidneys exhibits a 40.5% reduction compared to the control group. The utilization of selenium’s protective capabilities, present in the composite, demonstrates a reduction in the toxicity of silver.

The study authored by X et al. outlines the influence of polymers on drug release (timolol maleate) kinetics in nanoemulsion developed by an in situ gel formulation with applicability in the treatment of glaucoma. This study sought to enhance drug accessibility in glaucomatous conditions by incorporating varying proportions of the polymer Carbopol 934p, a polyacrylic acid polymer that exhibits a sol-to-gel transition in aqueous solutions when the pH exceeds its pKa of approximately 5.5, and it is extensively utilized in ophthalmology to improve pre-corneal retention in the eye. The NEI_5_ formulation maintained relatively stable effective drug levels in the ocular cavity for 24 h, and in vivo results demonstrated that the NEI_5_ offered superior sustained drug release compared to the conventional marketed dosage form. The timolol maleate nanoemulsion in situ gel formulation exhibited stability under storage conditions, showing no discernible alteration in appearance.

The study conducted by Vitus et al. highlights the production of a carbon-based bioscaffold (CHAK) utilizing carbon sourced from human hair and composite materials comprising konjac glucomannan (KGM) and agar by gelation, fast freezing, and ethanol immersion techniques. The developed CHAK exhibited comparatively moderate degradability (less than 50% after 28 days of incubation), substantial swelling (higher than 1000%), and exceptional water absorption capacity (higher than 90%). The 3AHC bioscaffold exhibited the highest electrical conductivity (21.14 ± 2.29 S/m) among the produced bioscaffolds. Finally, the biocompatibility assessment with Wharton’s jelly-derived mesenchymal stem cells (WJMSCs) indicated that the CHAK demonstrates compatibility, with over 90% cell survival on day 7. The results of this study demonstrated the viability of generating carbon from human hair as a material for the fabrication of a sustainable carbon-based bioscaffold for tissue engineering applications.

Yudaev et al., in their review paper, aim to delineate and rigorously evaluate research published in recent years regarding the utilization of polymeric dental nanoparticles as antibacterial agents across several domains of dentistry such as dental treatment, orthodontics, dental implantology, dental prostheses, and maxillofacial surgery regarding their antimicrobial efficacy. The breakthroughs outlined in the paper establish a new trajectory for the advancement of restorative dental materials and composites, with the objective of enhancing patients’ quality of life.

## 3. Conclusions

This Special Issue brings together a wide range of studies based on polymers with various applications in the biomedical field, ranging from cancer treatment, glaucoma, tuberculosis management, wound healing, the treatment of neuropsychiatric diseases such as schizophrenia, a reduction in bone tissue infection, and the development of materials with enhanced antibacterial, antifungal, or improved anti-SARS-COV-2 activity. Therefore, this compilation of articles encompasses a diverse range of research, elucidative of the richness of the topic [11].

The increased interest shown by renowned researchers from all corners of the world has led to this Special Issue having two more successive issues, now reaching Biomedical Application of Polymeric Materials III. I would therefore like to conclude this editorial by inviting you to be part of this Special Issue through your innovative research contributions in this broad and promising field.
